# More than a method: trusting relationships, productive tensions, and two-way learning as mechanisms of authentic co-production

**DOI:** 10.1186/s40900-021-00262-5

**Published:** 2021-05-31

**Authors:** Sarah E. Knowles, Dawn Allen, Ailsa Donnelly, Jackie Flynn, Kay Gallacher, Annmarie Lewis, Grace McCorkle, Manoj Mistry, Pat Walkington, Jess Drinkwater

**Affiliations:** 1 Centre for Reviews and Dissemination, University of York, York, YO10 5DD UK; 2 Patients in the Learning Health System PPI Group, NIHR Collaboration for Leadership in Applied Health Research and Care Greater Manchester University of Manchester, Manchester, UK; 3 School of Medicine, Leeds Institute of Health Sciences, University of Leeds, LS2 9JT Leeds, UK

**Keywords:** Co-production, Co-design, Participatory methods, Knowledge mobilisation, Patient involvement, Patient engagement

## Abstract

**Background:**

Knowledge mobilisation requires the effective elicitation and blending of different types of knowledge or ways of knowing, to produce hybrid knowledge outputs that are valuable to both knowledge producers (researchers) and knowledge users (health care stakeholders). Patients and service users are a neglected user group, and there is a need for transparent reporting and critical review of methods used to co-produce knowledge with patients. This study aimed to explore the potential of participatory codesign methods as a mechanism of supporting knowledge sharing, and to evaluate this from the perspective of both researchers and patients.

**Methods:**

A knowledge mobilisation research project using participatory codesign workshops to explore patient involvement in using health data to improve services. To evaluate involvement in the project, multiple qualitative data sources were collected throughout, including a survey informed by the Generic Learning Outcomes framework, an evaluation focus group, and field notes. Analysis was a collective dialogic reflection on project processes and impacts, including comparing and contrasting the key issues from the researcher and contributor perspectives.

**Results:**

Authentic involvement was seen as the result of “space to talk” and “space to change”. "Space to talk" refers to creating space for shared dialogue, including space for tension and disagreement, and recognising contributor and researcher expertise as equally valuable to the discussion. ‘Space to change’ refers to space to adapt in response to contributor feedback. These were partly facilitated by the use of codesign methods which emphasise visual and iterative working, but contributors emphasised that relational openness was more crucial, and that this needed to apply to the study overall (specifically, how contributors were reimbursed as a demonstration of how their input was valued) to build trust, not just to processes within the workshops.

**Conclusions:**

Specific methods used within involvement are only one component of effective involvement practice. The relationship between researcher and contributors, and particularly researcher willingness to change their approach in response to feedback, were considered most important by contributors. Productive tension was emphasised as a key mechanism in leading to genuinely hybrid outputs that combined contributor insight and experience with academic knowledge and understanding.

**Supplementary Information:**

The online version contains supplementary material available at 10.1186/s40900-021-00262-5.

## Plain English summary

We conducted a study exploring how patients could be involved in improving services using health data. This paper reports on the evaluation of that study. We collected different kinds of feedback throughout, including a survey of impacts on contributors, a focus group to reflect on what worked well and what could be done better, and also sharing thoughts throughout the study itself. We analysed this feedback together, to make sure that both contributor and researcher perspectives were considered equally.

We found that the successful co-production that happened during the study was the result of having ‘space to talk’ and ‘space to change’. Space to talk that meant we all shared our views and recognised each other as experts bringing equally important knowledge. Space to change meant that we acted on the knowledge shared, to change both the study and to change how we worked together.

We found that these themes occurred at multiple levels. They were partly achieved by the specific participatory codesign methods that were used, but equally or perhaps more important was the trusting relationship between the researcher and contributors, including openness to explore tensions. The wider systems that supported involvement, in this case the financial reimbursement for contributor time, were also as important to this as the things that happened within the study itself.

We recommend that future work creates ‘space to talk’ and ‘space to change’, and reports openly on how both contributors and researchers are affected by this.

**Supplementary Information:**

The online version contains supplementary material available at 10.1186/s40900-021-00262-5.

## Background

Driven by concerns about the gap between health research and practice, the field of knowledge mobilisation has developed to identify and evaluate ways to improve the translation of research evidence into improvements in care [1]. Current accounts of knowledge mobilisation emphasise the need for co-production of knowledge with the end knowledge users, in contrast to unidirectional pipeline accounts of knowledge being produced by researchers and then moved into practice [2].

Knowledge mobilisation activity is described as ‘mode 2’ thinking, which is the argument that collaboration between knowledge producers (academics) and knowledge users (those delivering or receiving health care) is required, rather than knowledge, referring to evidence, understanding and ideas about health care experiences, interventions, and services, being produced independently by researchers and then transferred to the knowledge users [3]. This approach is seen as necessary given the contested and dynamic nature of knowledge, which means it is not straight forward to transfer knowledge from one group to another, and that attempting to do so neglects the knowledge that the users themselves already hold. This recognises that there are different kinds of knowledge and expertise necessary to use to fully understand and improve health care This includes contextual knowledge about how services operate and experiential knowledge gained from living with and directly experiencing an illness or service [4]. It is argued that exploiting differences between kinds of knowledge can lead to greater insights [5] than would be possible alone. Knowledge mobilisation studies therefore explicitly aim to understand different types of knowledge, and identify successful means for surfacing, negotiating and integrating different ways of knowing.

Most work to date has focused on health and policy professionals as knowledge users, and patients remain a neglected and underutilised stakeholder group in knowledge mobilisation research [6]. Studies which have explored the role and impacts of patients in knowledge mobilisation settings (such as service improvement) have found that it can be fraught with difficulties specifically due to the challenges of reconciling different types of knowledge. Researchers may engage in “boundary defence” which refers to questioning the legitimacy of experiential knowledge in contrast to knowledge produced by academia [7].

This is consistent with findings in other areas of health research which report a lack of two-way knowledge sharing between patients and academics, with much patient involvement taking the form of consulting on specific aspects of research, for example to determine the acceptability of patient-facing materials, rather than engaging in dialogical knowledge exchange. This is despite accounts of authentic co-production focusing on the importance of allowing diverse forms of knowledge to interact [8, 9]. The present study aimed to understand patient involvement in health research specifically through a knowledge mobilisation lens that focused on successfully blending different ways of knowing and to provide a worked example of this process in action.

Research on effective co-production with patients has emphasised the need for dialogic processes which enable shifts in thinking and produce blended or hybrid knowledge outcomes [10, 11]. The extent to which traditional academic ways of working, with committees, meetings, and agendas, can enable this way of working has been called into question [12]. The range of models for patient involvement however remains limited [13] and formal group meetings remain the most common format [14]. These can tend to operate with predefined agendas which may restrict the potential for open dialogue, and there is a need to identify methods which enable co-production as an exploratory and generative process [15]. Tierney et al., in a critical review of involvement practices [16], argued that additional and/or creative activities may be expected in this field, yet are under explored to date, with accounts needed of what strategies can be employed to meaningfully enact collaborative working.

Design methods have increasingly been used in health research, moving from their original focus on technologies [17] to explore how design approaches can facilitate novel patient involvement in research [18], in quality improvement [19], and more recently knowledge mobilisation [20]. Collaborative design workshops may support the production of collaborative outputs as results of the discussions are immediately available for participants to engage with rather than being analysed and presented later by researchers [21], allowing for ongoing and active synthesis between ways of knowing. Participatory design is considered particularly useful for enabling co-creation of ‘boundary objects’ [22] which serve to represent different ways of knowing between different groups. Participatory codesign involves the use of physical and visual objects such as charts and maps [14]. These serve as ‘representational artefacts’ that facilitate a shared understanding and enable shared manipulation of materials [22]. These methods may therefore may be particularly valuable for eliciting different ways of knowing and blending knowledge. Such methods are also considered to more explicitly and inherently value experiential ways of knowing [22] and could provide a platform that encourages recognition and use of experiential knowledge.

This paper reports on the collective evaluation of the involvement practice and processes in a knowledge mobilisation research study which employed participatory codesign to generate ideas for patient involvement. There is a recognised need for more transparent and critical reporting of involvement. Tierney et al. [16] recommended that researchers publish more explicit accounts of how they defined service user involvement and their choice of methods for achieving this contribution, with the end goal of producing a ‘repertoire of practice’, and encouraging both methodological innovation and critical appraisal in the field. However, crucially this evaluation and reporting work needs to be done with patients/public, rather than only reported and evaluated from the perspective of researchers.

Evaluation and impact reporting have been contentious issues in involvement, as they are linked to instrumental or technocratic understandings of co-production which prioritise outputs of value to researchers [23]. The aim of the present evaluation was therefore to explore perspectives of the processes and impacts for both researcher and contributors, all of whom are co-authors of the paper.

### Aim

To evaluate, with contributors themselves, how participatory codesign methods contributed to the facilitation of sharing and blending different ways of knowing in a knowledge mobilisation research study.

## Methods

### Setting

The study was part of a Knowledge Mobilisation Research Fellowship, held by the lead author. The goal of the fellowship was to work with public contributors to design ways for patients, carers and members of the public to be involved in a Learning Health System (LHS). A LHS is a conceptual model of rapid data-driven improvement, suggested as a model of how health data, that is routinely collected by services, could become part of virtuous cycles of learning and feedback [24, 25]. Patients are intended to be key stakeholders in such systems, but there has been limited exploration into how this would be achieved, and a need to work collaboratively with patients themselves to consider how this should be done. The fellowship adopted a participatory codesign approach to exploring this with public contributors in the North West of England, UK.

Public contributors refers to members of the public with lived experience as patients, service users or carers who are actively involved in research as collaborators rather than as participants. The fellowship specifically involved collaboration with ‘expert’ contributors with a variety of experience of involvement in research, in recognition that this experience and expertise meant they had considerable insight into involvement, to support the generation of ideas about how involvement in a Learning Health System would look. Contributors were therefore recruited through the Fellow’s involvement networks. 11 contributors were sent an introductory email and person spec detailing the work and their expected contribution, including reimbursement and time requirements. All contributors expressed interest in joining the study, but three (two female, one male) had to withdraw their interest due to illness. The final group comprised of eight members, one male and seven females.

The codesign activities within the workshops included:

- Narrative methods: Reflecting on patient personas, narratives of exemplar patients based on published qualitative studies, to consider their user needs [26].
- Modelling methods: Drawing on studies that use future modelling and mapping to generate hypothetical examples [27, 28], contributors imagined how the system could look in practice and who would be involved, including considering an ideal future (Utopia modelling) and the worst possible outcome (Dark modelling)

The synthesis throughout the workshops was achieved visually and collectively, with contributors adding post-it notes, and the researcher using affinity mapping to summarise the discussions, compare and contrast key points, and agree priority messages. Rather than individual methods leading to discrete findings, the workshops were a cumulative and iterative process of refining earlier ideas through ongoing dialogue.

The methods were therefore collective, in that they occurred as a group with the contributors and researcher discussing them together, and iterative, in that we returned back to early discussions or revisited responses to the modelling and persona activities, consistent with the goals and ethos of participatory codesign approaches. Methods used and outcomes of these are discussed in more detail in the companion report (Knowles et al., under review).

Ten workshops were completed sequentially with the group of eight contributors, each lasting for three hours, and with subsequent further email and remote discussion regarding the study outputs. All workshops were attended by all contributors and the researcher, with the exception of workshop 3 which had 7 attendees (1 contributor absent) and workshop 7 which had 7 attendees (1 contributor absent).

The workshops therefore deliberately overlapped in content, but broadly followed this agenda:

- Workshops 1–2: Exploring what health data includes or excludes, drawing on the personas to think about the patients’ needs and experiences
- Workshops 3–4: Speculative modelling of what patient involvement in the system could look like and prioritisation of key aspects.
- Workshop 5–6: Mapping out a hypothetical patient-driven system, who would be involved, and what it would achieve
- Workshops 7–10: Reflection and synthesis to agree key learning for the study outputs.

### Evaluation data

Several forms of qualitative evaluation data were collected at multiple time- points throughout the study.

1. Survey: The Generic Learning Outcomes framework was developed in public engagement to capture potential impacts under five headings: Knowledge & Understanding, Skills, Attitudes & Values, Enjoyment, Inspiration & Creativity, and Behaviour & Progression [29]. This was used to frame the survey evaluation questions as it captured a wide range of potential impacts. Specific questions based on each heading were created by the researcher (SK) and provided to the contributors as a questionnaire with free text responses (Supplementary File 1). Contributors were asked to complete one after each workshop.
2. Documentary analysis of field notes taken by SK, a second researcher who attended five workshops as a note taker/observer, and of email discussions after the workshops.
3. An evaluation focus group, with six members of the study team (the lead researcher and five of the eight contributors), conducted after the 7th workshop. This was facilitated by a researcher experienced in Patient and Public Involvement (PPI) but external to the project (JD). The contributors were asked to reflect as a group on the questions ‘what went well?’, ‘what went not so well?’, and ‘what have I learned?’. These questions were deliberately broad to enable the contributors to focus on specific issues that mattered to them. These answers were discussed and responses recorded on post-it notes within the session. This provided data from photographs of sheets with contributor comments, and also a written account of the focus group provided by the external researcher one week later.
4. Through the process of preparing this paper, all authors have drawn on auto-ethnographic insights into the process.

### Analysis

There were three forms of analysis conducted regarding the evaluation of the co-design methods and the knowledge sharing that happened during the study:

1. Collective in-action analysis: Continuous reflection throughout the study itself, with both the researcher and contributors being collectively engaged throughout the study both as participants in the codesign process, contributors to the running and design of the overall project, and observers (data gatherers) of how all members worked together.
2. Retrospective thematic analysis of the collected qualitative data (survey responses, observational notes, post-meeting discussion emails, comments captured in the focus group, and the facilitator reflection account) in Nvivo, performed by the lead author, to identify key moments described as important and synthesise these into overarching themes.
3. Collaborative reflection in the final workshops, and in remote discussions after the end of the project, drawing on feedback collected throughout and sense-checking as a group the key moments and themes drawn from the thematic analysis. This involved SK presenting initial summaries of her understanding of the key findings, which were then further debated and discussed as a group to reach consensus or to identify areas where perspectives differed.

## Results

Our results are organised in the following way:

1. Description, with illustrative examples from the data, of the overarching themes that were agreed to underlie authentic knowledge-sharing in the project: ‘space to talk’ and ‘space to change’, which operated at three levels (methodological, relational, and institutional).
2. Description, with illustrative examples from the data, of how these themes manifested within the evaluation process itself.
3. Description of how these themes contributed to the creation of hybrid knowledge outputs in the main project.

### Part one: overarching themes

Collective reflections on the key processes in the study were synthesized into two overarching principles “space to talk” and “space to change” (Table 1). These principles were found to operate across three levels. Firstly, in the codesign methods employed. Secondly, in the relationship between the researcher and the contributors. Thirdly, in the institutional processes of how involvement was financially supported. The contributors themselves considered the second and third levels to be as or more important than the first level, the design methodology.

**Table 1:** Space to Talk and Space to Change across different levels

	Space to talk	Space to change
Design Methods	- Workshops not meetings. - focus on visual recording and sharing to provide a shared conceptual space for discussions. - Prioritising experiential knowledge so that it is recognised as expert knowledge. Survey feedback: “*The mind maps on flip charts are good as I like the free-thinking/mapping side of idea generation. There needs to be more visual, illustration in research and academia. It can be very dry and formal when it doesn’t need to be”*	- Iterative and reactive - Use of prompts and templates to invite critique and reflect changes made. - Collective ‘real time’ synthesis. Evaluation notes: “*the example of writing on the board and seeing how your words were being listened to, built on and “having an impact” felt good and reinforced their involvement … As they had done it as a big group everyone’s ideas were together”*
Relationship between contributors and researcher	Recognising and explicitly inviting public participants as equal experts Constructive tensions and disagreements deliberately explored Evaluation notes: “*The group attributed the good group bonding to [researcher] creating an open and transparent space in which they were all respected and as a result respected each other.”* Contributor email: “*I think it’s really important to emphasise that there were disagreements which weren’t always resolved, but that we did try very hard to come to a consensus and generally succeeded … the diversity of opinions was a crucial part of the discussions.”* *Contributor email: “we shouldn’t underplay the disagreements … The creative tensions were what made our work distinctive and, in my opinion, more valuable”*	Willingness to adapt the study aims in response to the contributors. Willingness of all to change minds in response to others. Sharing responsibility to collaboratively generate new solutions as well as critique existing models. Evaluation notes: *“Facilitator has to be a ‘risk taker’ and be open to ideas that are not anticipated or expected – too often researchers want to know the outcome from the start; [researcher] had to have ‘trust’ in outcomes from workshops that are not pre-planned; becomes about trust and not about power”* Workshop Six observations: “*Consensus that people have been open minded enough to change their views – there has been a flexibility and adaptability to new ideas.”*
Institutional processes	Providing cash reimbursement enabled attendance for some contributors and reinforced the fact that their input was genuinely valued. Contributor email: *“Some people literally could not afford the travel and opportunity costs of attending a meeting. Then there are the social/psychological dimensions of feeling that your contribution is not valued or taken seriously”*	Changed reimbursement in response to feedback – demonstrated willing to change way of working to respond to needs Survey feedback: “*It has been really good to see [researcher] actively consulted the group about payment and has listened to our frustrations”*

‘Space to talk’ refers to the need to create space for dialogue by explicitly recognising the importance of ongoing contributions from both the researcher and the contributors. This dialogue includes space for both agreement and disagreement, seeking to compare and contrast different ways of knowing in an open way. ‘Space to change’ refers to both willingness to change personal views or reach compromises as an individual and a group, and, at the project level, change in the work that was done. This involved transparently capturing how differences produced joint understanding or outputs, which were emergent and represented change from how the project was originally conceived. Together, these spaces led to contributors evaluating the study positively, as an example of authentic involvement.

These spaces occurred at three different levels in the study:

1. *Methodologically, through use of co-design methods:*

The specific design methods used were on the whole valued more by the researcher (although some contributors equally valued the specific methods as facilitating engaged discussion) as a way of accessibly presenting academic or conceptual knowledge in a way that enabled interrogation by the contributors. However, both researcher and contributors equally valued the openness and joint generation of ideas achieved through the methods of visual recording and real-time synthesis. The contributors also often expressed their ideas as visual descriptions. A professional illustrator was invited to the final two workshops to produce images that could better capture the meaning that contributors wanted to express.

Some contributors commented toward the end of the study that they would have preferred a more open process from the beginning, without the use of specific tools or design templates. The researcher and some other contributors however argued that this ‘blank slate’ would have made it more difficult to meaningfully grapple with the academic concepts presented, which was necessary in order to then deconstruct the academic model from their own perspective. Codesign methods can therefore provide a helpful scaffold, supporting the contributors to take responsibility as both ‘agitators and ideators’, critiquing existing understanding but then proposing solutions or new ways of working. The results demonstrate however that no single method will be valued by all contributors.

2. *Relationally, through the ways in which the contributors and researcher worked as equal partners:*

**Fig. 1:**
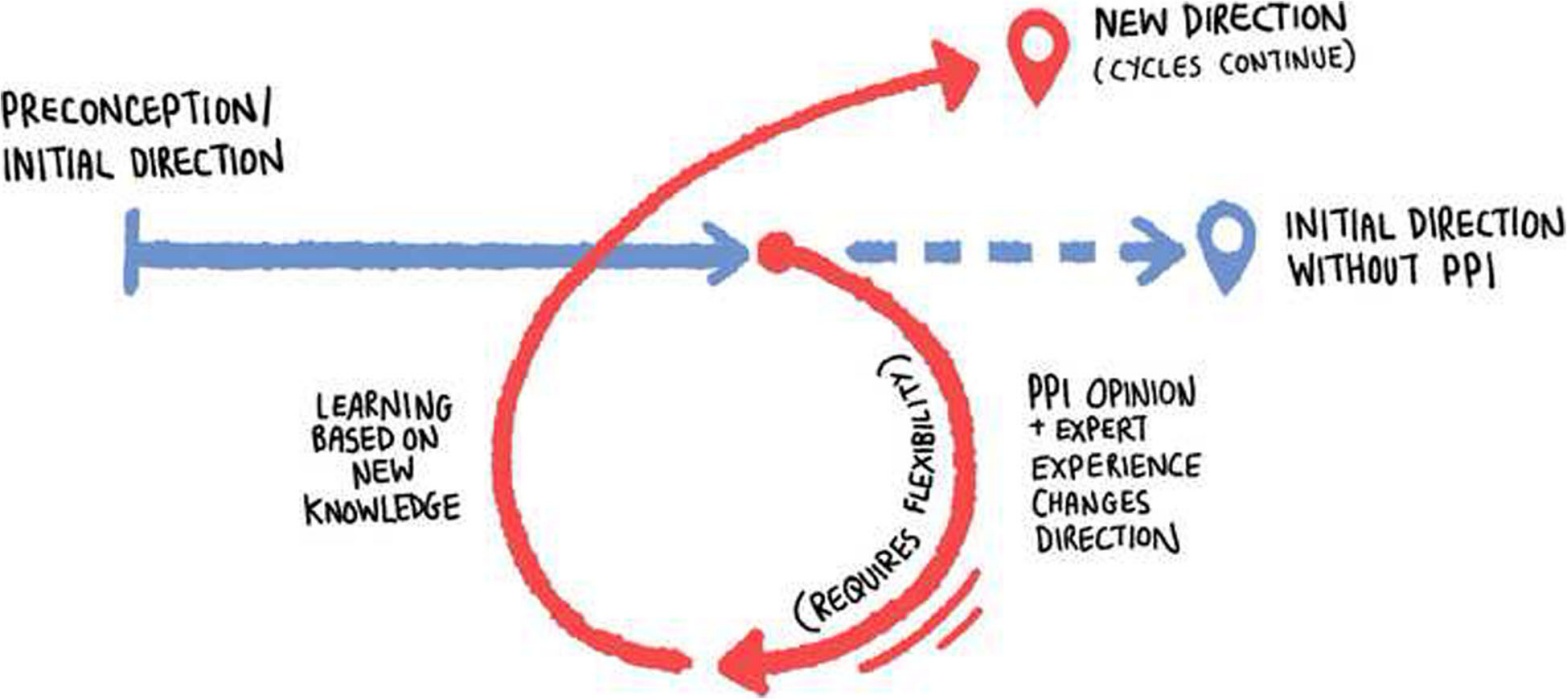
Illustration: Contributors' view of how PPI changes projects

The contributors emphasised that the relationship enacted in the workshops was more important than an individual method. Drawing on their past experiences, they argued that any method could be used either authentically or inauthentically depending on the individual researcher’s approach. The contributors especially emphasised the importance of the researcher adapting the study in response to the contributors’ feedback (Fig. 1).

**Fig. 2:**
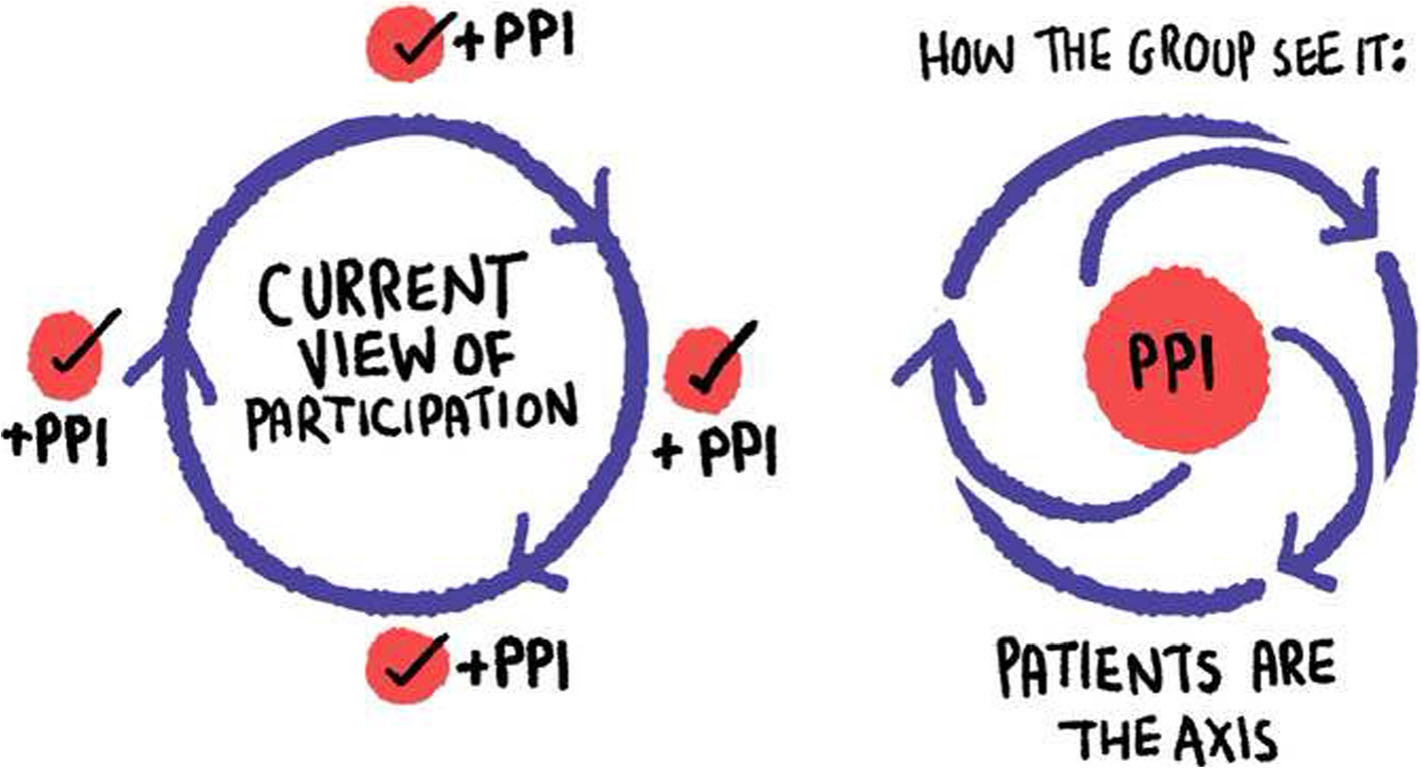
Illustration: Contributors view of how the project changed

**Fig. 3:**
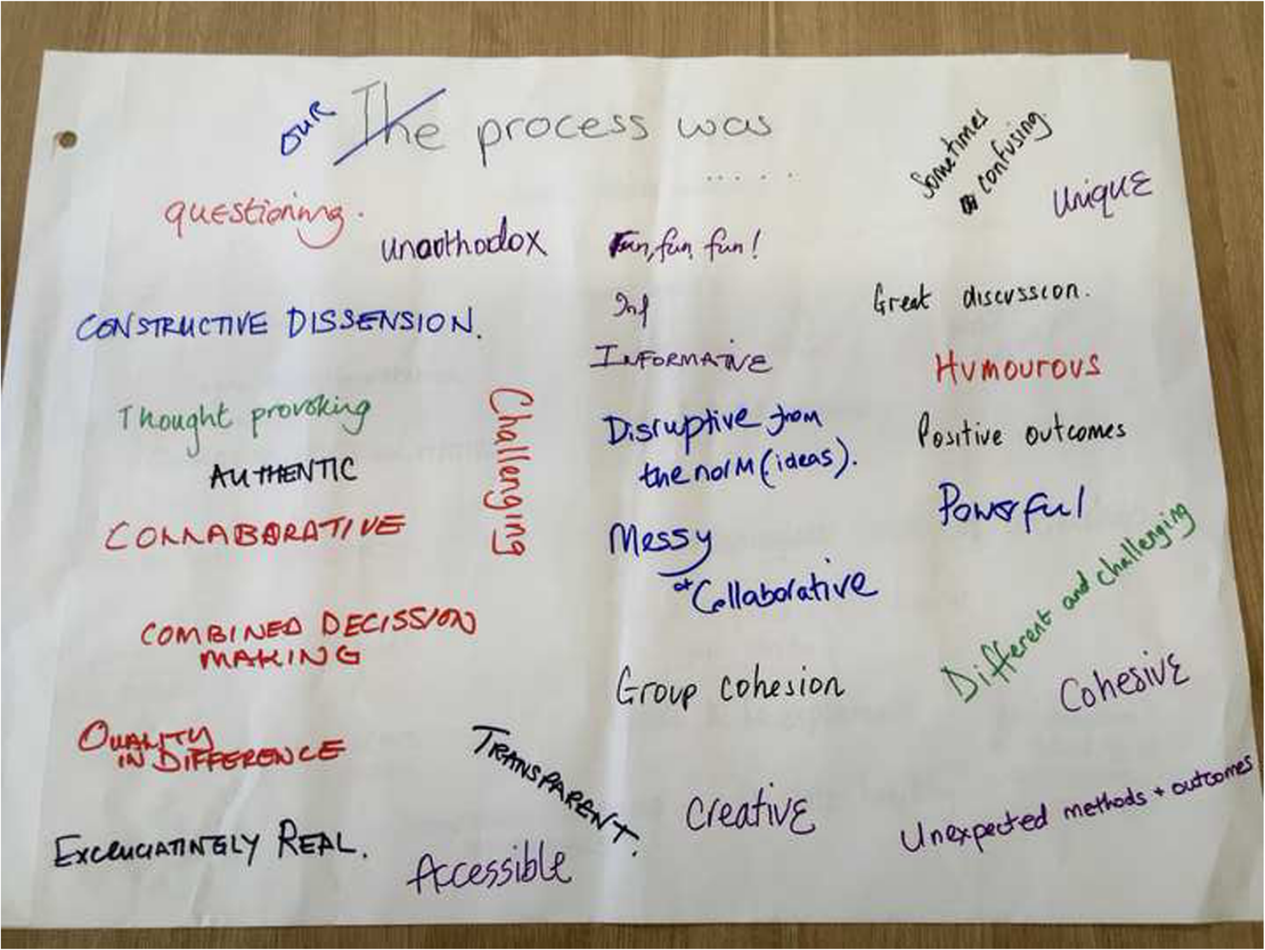
Workshop Feedback - Group reflections on the collaborative process

The contributors input fundamentally shifted the aims of the research over the course of the study. It moved from identifying opportunities for patient involvement in existing Learning Health System models, to more radically reimagining a “patient-driven” LHS, and more deeply interrogating the concept of “data” for guiding improvements (Fig. 2). Although this led to novel and conceptually rich findings, it was a source of anxiety for the researcher during the process, given the uncertainty about what ‘outcomes’ would be available to report, and also a concern that deviation from the planned protocol would be reviewed negatively by peers and supervisors. The contributors however greatly valued her willingness to work with the disagreements and repeatedly emphasised the importance of ‘productive tension’ as a critical learning mechanism in the collaborative work (Fig. 3).

3. *Institutionally, through the reimbursement process supporting the involvement work:*

While the researcher’s original conception of the evaluation focused on activities ‘in the room’ referring to interactions within the workshops, it became clear that contributors evaluated the process as a whole, including ‘beyond the room’ factors such as how practical issues of reimbursement were organised. At the start of the project, the researcher advised that payment would be made through the University’s claims systems, where payments are made within 4–6 weeks into the contributor’s bank account. The contributors responded negatively to this, pointing out that this significant delay was contrary to the stated aims of valuing contributor input. In addition, some contributors would be unable to attend without cash reimbursement on the day and so be excluded. In light of this, the researcher, with support from the CLAHRC director, negotiated the processes within her faculty and was able to provide cash reimbursement on the day of each workshop.

Through the evaluation, this emerged as a significant moment for the contributors. It was a concrete example of the researcher recognising the barriers that needed to be overcome for contributors to be present, in addition to responding to the critical feedback to make changes to how involvement was done. This laid a foundation of trust for the interactions within the workshops, establishing that contributors’ input was viewed as essential and so barriers to access would be tackled, and demonstrating responsiveness that recognised the legitimacy of contributor concerns.

### Part two: space to talk and space to change within the evaluation

Tensions between researcher assumptions and contributor perspectives emerged in the process of the evaluation focus group, as the contributors were focused on what the researcher had learned from the experience, rather than considering what they had learned during the process. The researcher was upset by this, as she interpreted that it meant contributors had not gained anything from the experience, and so felt anxious about whether she had provided them with any learning. Further discussion demonstrated that some contributors did perceive explicit learning to have occurred (both around the topic of health data and improvement and also new learning regarding the design methods employed). However, this difference – whether new learning had occurred or not – was itself considered a positive and authentic outcome by the contributors, as it reflected the diversity of the group rather than there being a consistent and uniform impact that was the same for all.

Where contributors did refer to learning, this was primarily described as being through relational openness in the group, rather than learning ‘from’ in an exchange of expertise:

*“One example of sharing learning was the Stakeholder mapping exercise. The group said that [researcher] had told them things about the different stakeholders that they didn’t know and were surprised about. Without [researcher’s] involvement at this stage they would have had different, and they admitted potentially unrealistic, expectations of the different stakeholders. Therefore, [researcher] opening up to them and contributing to the group were both essential. They described this safe environment and the ability to open up as “the trust between you” (Evaluation Focus Group Account)*

The researcher’s initial unhappiness that she had “failed” to help contributors was notable. It revealed an assumption that successful involvement would lead to outputs *from* the researcher *to* the contributors, exposing an expectation of some unidirectional exchange, whether of skills, experiences or knowledge. This distinction was also reflected in the survey, of which only 8 were completed, by 4 contributors. The contributors who completed the forms tended to reflect on the process as a whole rather than relating feedback to individual workshops. The survey had been put together by the researcher, rather than being decided on collaboratively. Contributors reflected that they struggled with the questionnaire as it did not adequately reflect the process they felt we were engaged in, as it described discrete impacts ‘on’ them, rather than being about collective and interactive learning.

The researcher discussed her anxiety with the group, expressing her fear that it brought into question her own expertise and contribution to the progress of the study. The contributors and researcher explored this tension together, and arrived at agreement that the researcher’s contribution was best understood as a “*catalyst*” for the discussions. This could lead to new and hybrid knowledge, rather than being an exchange of knowledge from one group or individual to another. This two-way interaction was valued by contributors, as it recognised and depended on their own expertise and knowledge, rather than assuming they would be passive receivers in the co-design process. It was also considered positively by the researcher, who felt this framing acknowledged her effort in supporting the group discussions. The initial tensions experienced around lack of reported learning were therefore resolved through understanding both the contributors and researcher need to have their expertise and contribution recognised as supporting two-way learning.

*“I think people do acknowledge that they learned something - developed their views - both because of you and because of each other … This is not a 'you and us' situation. Once we started working together we become a group - so we learned together.” (Contributor feedback)*

*“Your [researcher] changed way of looking at things was an ‘impact’ of the project” (Focus Group Written Feedback)*

Conducting the evaluation meeting as a focus group outside the regular workshops had in itself revealed tensions between a focus on two-way learning compared to more traditional evaluation. The researcher (SK) invited an ‘independent outsider’ (JD) to facilitate the focus group and give the contributors a separate space in which to express their views. However, the contributors themselves felt that this suggested that they were separate from the researcher – the subjects of the process - and wanted the evaluation to be a mutual discussion. A key part of this was for them to have the opportunity to ask questions of the researcher and for there to be progress in understanding the issues raised through interaction (including through identifying areas of disagreement). The contributors framed the evaluation much more about evaluating how they and the researcher had worked together, rather than evaluating ‘the project’. The evaluation process was therefore modified, with the contributors having one hour of independent feedback with only JD present, but then a second hour talking together with SK.

An unexpected outcome for the researcher was that the contributors actively asked for feedback on how she felt in their interactions, contrary to the idea that the researcher was a separate facilitator who was responsible for those interactions. This gave the researcher the opportunity to express some of her own anxieties, leading to changes in how the group gave her feedback in later sessions.

*“[researcher] said she was never sure if she was supporting the group enough. The group reflected that they felt really well supported, that the level of contact and openness (email, text message) were great. However, they also reflected that they don’t do the same thing for [researcher]. The group asked each other whether anyone had ever sent an email after a meeting to say how the meeting was and check in with [researcher] about how she was. They hadn’t really realised that [researcher] might be feeling like this and need this support and they agreed this was something that they should try and do in the future.” (Evaluation Focus Group Account)*

Overall, the evaluation demonstrated the value of active two-way discussion to inform ongoing working relationships, rather than separate or independent conversations or surveys.

The evaluation itself therefore demonstrates the importance of the two themes. "Space to talk" meant openly sharing experiences together as equal partners, including the researcher sharing her own anxieties as a collaborator ‘within’ the group and discussing these openly, rather than reflecting on separate surveys or discussions. "Space to change" was evident both in changing the evaluation process based on contributor feedback (with the researcher joining the focus group) and in the consequent focus of the contributors on how the way we worked together could be changed to be better (such as checking in after sessions).

### Part three: producing hybrid knowledge

Creating "Space to talk" and "Space to change" led to hybrid outputs that reflected a merging of different ways of knowing and understanding. The study findings were a merging of insights from the contributors about what mattered to them, with an academic understanding of health data and improvement. This included drawing on theoretical constructs which were perceived to add value in terms of understanding or communicating the key findings. In some cases this was about divergence between patient and academic ways of knowing. For example, a significant discussion was held around what ‘health data’ meant, and whether researcher conceptualisations of data would exclude the more experiential and dynamic information that patients considered most important to improving care. The discussion of what data would include or excluded led to novel academic insight, around how the theory of epistemic injustice could be considered to apply specifically to health data, due to the question of whether epistemologically different ways of knowing would be captured or excluded by data. The sharing of knowledge was a two-way process, but with the knowledge output then being greater than the sum of its parts. Rather than an additive conclusion, there was an interaction between the researcher’s theoretical knowledge and the contributors' experiential and contextual knowledge which led to a novel finding that was different to what either group would have produced independently.

In other aspects of the study there was notable convergence between academic understanding and contributor perspectives. For example, the contributors suggested three patient roles that could occur in the system, which it became apparent matched closely with the concept of three types of transparency (informational, participatory, and accountability) described in the literature on health data use [30]. This indicates a synergy in terms of the contributors’ priorities and the issues being explored in the academic literature, but with the contributors approaching this issue in a different way. Specifically, the public contributors conceptualised actual activities that patients could lead on that would enact these transparent ways of working. This produced a hybrid finding, with the conceptual framing of transparency found in the literature translated into practical and active representations.

The study outputs themselves also reflect a hybrid process, representing a shared report of the study, with the study paper for example explicitly reporting the changes contributors had made, rather than providing a more traditional linear report of the original aims and final interpretation.

## Discussion

The metaphor of ‘space’ is consistently used in accounts of co-production with patients [31]. This has included referring to ‘exploratory social spaces’ [15] 'knowledge spaces’ [8], ‘experimental spaces’ [12]. Bryant and colleagues [32], again in a collective evaluation of involvement, described the importance of ‘creating space’ both within individual attitudes and in making time for changes to occur. We hope that ‘“space to talk” and “space to change” acknowledges this previous literature but also express in plain language what ‘space’ means in practice for both contributors and researchers. The definition of spaces used particularly aligns with Abma and Broerse’s conceptualisation of core participatory mechanisms being those that enable ‘dialogue and iteration’ [13]. The present study has presented an analysis of how these can be enacted, and drawn attention to how methodology is only one component of realising these goals. This is consistent with research emphasising that participatory collaboration is negotiated through relational means, and shows this occurs both in terms of individual relationships with research partners, but also as an institutional process of supporting involvement.

The link between talking and changing can be a process of disagreement and divergence in perspectives. The present study demonstrates that these differences should be welcomed as core mechanisms of action in co-production, as they surface differences in understanding or ways of working, and enable active and collective exploration of these to find ways to move forward. In a study of values underpinning involvement, Snape and colleagues reported that tensions due to differences in perspective were viewed as both inherent to involvement work and a valuable means of stimulating critical debate [33]. The contributors themselves emphasised during the review of study outputs, including the academic papers, that we should not try to present a clean picture but should expect and acknowledge tensions since these can provoke valuable changes. This transparency in reporting is necessary to emphasise that the process of disagreement can be productive for both researcher and contributors, with contestation a key process in acknowledging differences [34] and enabling space for them to discussed.

There remain points about the project where we did not achieve consensus, but retain differing opinions, both between academic and contributors and between contributors themselves. An example is the terminology employed in this report itself, specifically the choice of the word “spaces”. Some contributors disliked the term as it evoked the idea of ‘safe spaces’, which they argued would obscure a necessary acknowledgement of risk and disruption, in contrast to safety. A more apt term may be to consider the need for ‘brave spaces’ [35] where challenging encounters and negotiations can occur. It is necessary to consider how such encounters can be supported, to enable both contributors and researchers to engage openly with what can be difficult processes. This may also require bravery from research funders and directors, to allow for uncertainty in how projects develop and trust that iterative working will produce relevant outputs.

It should not be underestimated that adaptive processes of ‘productive tension’ and ‘constructive dissent’ can be challenging for researchers to engage with. This can be due both to individual reservations and due to the misalignment of this way of working with traditional ways of completing and reporting projects. This need to trust in unexpected outcomes can therefore feel threatening, although evidence also suggests it can be rewarding [36]. In the present study, the researcher had relatively more freedom to engage in changes to her ways of working or to her original conceptualisation of the study as the project was part of an individual fellowship. The structures of academic funding and progress can be prohibitive to efforts to work iteratively and responsively [37] but making changes in response to patient input is a crucial part of achieving authentic co-production. “Space” was chosen as the descriptor deliberately to contrast the need for openness with the typically closed spaces within academia, with academic structures, including project governance, reporting and funding arrangements, which all contribute to limiting the space for adaptation and space to explore divergent views [34, 37, 38]. The responsibility for creating such spaces should be recognised as the responsibility of wider academic systems as much as the responsibility of individual researchers or teams.

The study has the following implications, firstly for involvement practice and secondly for involvement evaluation.

### Implications for involvement practice

Design methods were valuable in the study, but proved to be only one element in achieving an overall process supporting two-way conversation and collective iteration. The attitude of partnership working was considered more important, and contributors noted that methods could be employed in a way which lacked the corresponding respect for partnership and commitment to two-way learning. Individual methods or techniques should therefore not be viewed as sufficient to achieve partnership working (it should also be acknowledged that even formal academic meetings can achieve partnership, if relational elements are attended to [39]). This is consistent with warnings from the literature that methods alone are not enough to facilitate co-production in the absence of genuine dialogue and reflection [34] and that codesign techniques themselves do not guarantee collaboration in the absence of reflection and dialogue [34].

The emphasis on the relational way of working as a key mechanism is consistent with research which has emphasised how relationships should be prioritised in co-production [11]. This study expands this further to argue that processes ‘beyond the room’ need to be considered as carefully, as they impact as much on involvement as the activities which are considered by the researchers themselves to be ‘the’ involvement activity. Although reimbursement practices are acknowledged as important in best practice guidance, the issue is rarely deeply interrogated in the evaluation literature (a notable exception being the paper by Richards and colleagues [40], who are themselves patient contributors, and a reflective account from a Community Participatory research about how neglecting the financial burden of research can be the results of unexamined privilege [41]). Methodological literature has considered how design artefacts can function as boundary objects. Academics should recognise that practical processes such as ‘how reimbursement happens’ also function as boundary objects. These are used by public contributors to discern how authentic the researchers’ expressed commitment to them is and how much their experience and knowledge is genuinely valued. This can be overlooked in practice. Far from being separate administrative issues, these processes provide, for contributors, symbolic as well as financial indications of how much they are valued (or not) by Universities.

The findings suggest that researchers wishing to produce hybrid knowledge outputs with patients should focus on explicitly surfacing and exploring epistemological disagreements. Staley and colleagues have demonstrated the usefulness of training for contributors which helps them consider explicitly how they share their experiential knowledge, and are developing mirror training for researchers [42]. We found that inviting an illustrator to capture and portray the contributor comments was particularly useful in helping them to express their priorities. Future studies should consider adopting this approach, being mindful of the additional costs required (for example planning for this in grant applications).

The role of the researcher as a ‘catalyst’ for such discussions is worthy of more exploration, for example considering how researchers can best facilitate such processes, if researchers, contributors, or others are best placed to act as facilitators, and how facilitation can include appropriate choice of methodologies but also relies on communication, reflection, and relationship building [43]. In the present study, SK had worked with several of the contributors previously. This helped in achieving a trusting relationship that meant disagreements could be productively explored. The contributors were also more willing to share personal lived experiences to inform the work based on that trusting relationship. Within academia, there is a focus on “refreshing” (recruiting new public contributors) rather than building on more long-term relationships, as this is perceived to be helpful in gaining new views. Future work should consider both how ‘productive tension’ can be managed in new collaborations, and in addition recognise the benefits of supporting sustained relationships between contributors and researchers.

Both a contributor and researcher co-author emphasised the similarities of the findings to the principles of Action Research. In particular, Action Research deliberately anticipates and acknowledges unexpected outcomes [44]. Given the emphasis in knowledge mobilisation on co-production of knowledge, the Action Research literature may be particularly useful to help researchers understand how such co-production offers unique advantages and challenges compared to traditional research approaches [45]. An Action Research perspective would agree with the findings presented here, that co-production occurs not at discrete stages of a project (occurring through a particular method such as co-design) but is achieved through equal collaboration throughout and about a project.

### Implications for evaluation

The findings demonstrate that evaluation of involvement needs to be careful not to assume a one-way process of learning from researcher to contributors. Instead, two-way learning and change should be anticipated and reported. Cockcroft and colleagues have similarly suggested that changes in researcher knowledge or understanding are an important part of involvement, but typically unreported in accounts [46], and Staley has argued that involvement often produces impact through how it affects researcher’s understanding and actions [47]. This paper has attempted to address this under-reporting and provide an honest account of where researcher assumptions were challenged, and acknowledge the significant learning and change the researcher experienced.

The contributor co-authors observed that on an earlier draft, their own feelings were referred to, but the researcher’s reactions were described without reference to emotion. For example, the paragraph in the results section about the researcher’s anxiety about her contribution was originally described in terms of “The researcher found this implication disconcerting” rather than stating the emotional impact (“The researcher was upset”). It was agreed that researchers need to be transparent about the feelings they have during involvement, both to make descriptions more equal across contributors and researchers, and so that the emotional impacts can be acknowledged and understood [48].

A focus on how different perspectives are compared and contrasted, and whether these lead to agreed hybrid outputs, indicates a need to evaluate the processes employed to elicit differences in perspectives and manage these differences constructively, rather than only evaluation of discussion outputs [49]. This is likely to lead evaluations to consider the relationships that exist and are developed between researchers and contributors, rather than providing a simplistic account of any single method which can achieve such impacts [43]. In particular, evaluation is necessary to better understand how disagreement and tension become “productive” dissent, and be open to reporting the impacts on researchers and contributors when such tensions are not resolved.

The findings also illustrate that researchers should not assume that the same impacts will occur for everyone on every occasion. Evaluation should be flexible and recognise the differences that contributors bring, both in terms of their own ‘baseline’ of experience or knowledge, and the kind of learning that they want to happen. Evaluations should be negotiated with contributors to agree impacts that are meaningful to them, including co-designing or agreeing the best ways to capture feedback on these.

### Limitations

The study did not explore whether the principles of ‘talk and change’ applied to direct knowledge mobilisation activity, as the study explored hypothetical models of involvement. It is therefore necessary to explore whether the same principles underpin the application of knowledge mobilisation activity.

We have reflected on how the relatively independent nature of the fellowship allowed the changes to be made, and questioned whether wider projects can adopt this approach. This is an important area of exploration for future work.

The paper reports on a single study with only one researcher involved. This allowed in depth exploration with multiple sources of data and particularly supported the collaborative reflection on evaluation with contributors, but comparative case studies which explore differences in researcher approaches and different study contexts will be useful in further understanding the core processes underpinning ‘authentic’ co-production.

## Conclusion

This collaborative evaluation found that across the different levels of methods, relationships, and institutional processes, the project provided *space to talk* and *space to change* in a way that led contributors to describe it as authentic co-production. The results demonstrate that the path to this can be unpredictable and anxiety-inducing for the researcher. However, the difficulties encountered were considered by both the researcher and contributors to provide productive tensions which, if explored, could provide opportunities for improved ways of working together and for the creation of genuinely hybrid knowledge outputs that merge academic and experiential ways of knowing.

## Supplementary Information

**Additional file 1.**

## Data Availability

Materials used and visual data produced within the workshops are available from the lead author on request. Individual and group evaluation data is not available due to confidentiality.

## Abbreviations

LHS: Learning Health System
GLO: Generic Learning Outcomes
