# Supplementary material for: More than a method: trusting relationships, productive tensions, and two-way learning as mechanisms of authentic co-production
Source: Res Involv Engagem. 2021 May 31;7:34. doi: 10.1186/s40900-021-00262-5 (PMC8165763; doi:10.1186/s40900-021-00262-5)
Supplement: Supplementary file 1 — Additional file 1. [file 40900_2021_262_MOESM1_ESM.docx]

Survey questions based on the Generic Learning Outcomes:

1. Has your knowledge and understanding of the topic changed? This can include: learning something new, deepening understanding, making sense of something, seeing links between things.

2. Have you learned any new skills? This can include: knowing how to do something new, changes in communication or social skills, new skills in managing information.

3. Have your attitudes toward the topic changed? This can include: changed opinions about the topic, people or organisations, having more positive or more negative perceptions, increased or decreased motivation to learn more or be involved in the topic.

4. Did you enjoy the activities? This can include: being surprised or inspired, being able to explore, innovate or be creative, having fun, experimenting with something new.

5. Has your activity or behaviour changed? This can include: intention to be involved in this work or work like it, change in how you will be involved or want to be involved, progression in feeling more able to be involved.
